# Functional analysis of an unusual type IV pilus in the Gram‐positive *Streptococcus sanguinis*

**DOI:** 10.1111/mmi.13237

**Published:** 2015-10-27

**Authors:** Ishwori Gurung, Ingrid Spielman, Mark R. Davies, Rajan Lala, Peter Gaustad, Nicolas Biais, Vladimir Pelicic

**Affiliations:** ^1^ MRC Centre for Molecular Bacteriology and InfectionImperial College LondonLondonUK; ^2^ Department of BiologyBrooklyn College of the City University of New YorkNew YorkNYUSA; ^3^ The Wellcome Trust Sanger InstituteHinxtonCambridgeUK; ^4^ Australian Infectious Diseases Research CentreThe University of QueenslandBrisbaneQueenslandAustralia; ^5^ Department of MicrobiologyOslo University HospitalOsloNorway

## Abstract

Type IV pili (Tfp), which have been studied extensively in a few Gram‐negative species, are the paradigm of a group of widespread and functionally versatile nano‐machines. Here, we performed the most detailed molecular characterisation of Tfp in a Gram‐positive bacterium. We demonstrate that the naturally competent *S* *treptococcus sanguinis* produces retractable Tfp, which like their Gram‐negative counterparts can generate hundreds of piconewton of tensile force and promote intense surface‐associated motility. Tfp power ‘train‐like’ directional motion parallel to the long axis of chains of cells, leading to spreading zones around bacteria grown on plates. However, *S* *. sanguinis* Tfp are not involved in DNA uptake, which is mediated by a related but distinct nano‐machine, and are unusual because they are composed of two pilins in comparable amounts, rather than one as normally seen. Whole genome sequencing identified a locus encoding all the genes involved in Tfp biology in *S* *. sanguinis*. A systematic mutational analysis revealed that Tfp biogenesis in *S* *. sanguinis* relies on a more basic machinery (only 10 components) than in Gram‐negative species and that a small subset of four proteins dispensable for pilus biogenesis are essential for motility. Intriguingly, one of the piliated mutants that does not exhibit spreading retains microscopic motility but moves sideways, which suggests that the corresponding protein controls motion directionality. Besides establishing *S* *. sanguinis* as a useful new model for studying Tfp biology, these findings have important implications for our understanding of these widespread filamentous nano‐machines.

## Introduction

Type IV pili (Tfp) are long, thin and flexible surface‐exposed filaments found in Bacteria and Archaea (Jarrell *et al*., 2013; Berry and Pelicic, 2015). Tfp are homo‐polymers composed of subunits synthesized as precursors with a characteristic class III signal peptide (Szabó *et al*., 2007), an N‐terminal motif (Giltner *et al*., 2012) consisting of a hydrophilic leader peptide followed by a tract of predominantly hydrophobic residues that form an α‐helix named α1N (Craig *et al*., 2004). Subunits are assembled in filaments by a complex machinery that includes distinctive proteins (Pelicic, 2008). These defining features are shared by a group of filamentous nano‐machines called type IV filaments (Tff), which mediate an astonishing array of functions (Berry and Pelicic, 2015). Tff are almost universal in prokaryotes as genes encoding the above characteristic proteins are found in more than 1,800 species, spanning almost all phyla of Bacteria and Archaea (Berry and Pelicic, 2015).

Much of our understanding of Tff biology comes from studies of bacterial Tfp in a few Gram‐negative human pathogens belonging to the phylum Proteobacteria (Pelicic, 2008), where Tfp have been studied in depth because they are key virulence factors. These studies have defined two sub‐classes of Tfp (Giltner *et al*., 2012). Tfpa (i) are composed of pilins with shorter leader peptides sharing substantial sequence conservation in α1N (Berry and Pelicic, 2015), (ii) are synthesized by a machinery consisting of 15 conserved proteins (encoded by genes scattered throughout the genome) (Pelicic, 2008; Berry and Pelicic, 2015) and (iii) promote a wider array of functions, including a form of surface‐associated motility known as twitching motility (Mattick, 2002) and DNA uptake in naturally competent species (Chen and Dubnau, 2004). These additional properties are a direct consequence of Tfpa's ability to retract (Merz *et al*., 2000), which is powered by the PilT ATPase (Satyshur *et al*., 2007), and generate huge forces in the piconewton (pN) to nN range (Maier *et al*., 2002; Biais *et al*., 2008). In contrast, Tfpb (i) are composed of less conserved pilins with longer leader peptides (Berry and Pelicic, 2015), (ii) are synthesized by simpler but more heterogeneous machineries of 10–13 proteins (encoded by genes that cluster together) (Pelicic, 2008) and (iii) have not been shown to retract and generate force.

There are important gaps in our understanding of Tfp biology and the establishment of a Gram‐positive model, inherently simpler because of the absence of an outer membrane, would represent a promising new research avenue. Unfortunately, although Tfp biogenesis genes are widespread in Gram‐positive species, these filaments have been scarcely characterised so far (Melville and Craig, 2013). Tfpa have been studied to a limited extent in Clostridia, *i.e. Ruminococcus albus* where they might mediate binding to cellulose (Rakotoarivonina *et al*., 2002) and several *Clostridium* species exhibiting a form of motility on agar plates consistent with twitching motility (Varga *et al*., 2006). Gram‐positive Tfpb have been even less characterised with only one report in *Bifidobacterium breve* (O'Connell Motherway *et al*., 2011). No in‐depth functional analysis has been performed in any of these species because they are genetically intractable. A Gram‐positive species expressing Tfp with easy genetics would thus represent an important step forward. To us, *Streptococcus sanguinis*, a commensal of the human oral cavity and opportunistic pathogen causing infective endocarditis (Que and Moreillon, 2011), seemed an ideal candidate. Indeed, in studies published 30–40 years ago, it was reported that *S. sanguinis* isolates are often piliated and exhibit a form of motility on agar plates consistent with twitching motility (Henriksen and Henrichsen, 1975; Henriksen and Eriksen, 1976; Gaustad and Froholm, 1984). However, these studies were published before the molecular biology era, and no genetic evidence was ever produced to confirm that *S. sanguinis* expresses Tfp. Strikingly, this species has since become a Gram‐positive workhorse for molecular genetics (Xu *et al*., 2011) because its competence for natural transformation, which is a property common in *S. sanguinis* isolates (Gaustad, 1979; Gaustad *et al*., 1979), can be readily induced using a synthetic competence‐stimulating peptide (CSP) (Håvarstein *et al*., 1997).

Therefore, in the present study, we have used this genetic tractability to our advantage to embark on an in‐depth study of Tfp biology in *S. sanguinis*. Using an approach combining genomics, molecular genetics, biochemistry and biophysics, we have sought to answer the following questions: (i) does *S. sanguinis* express Tfp, (ii) what are these filaments composed of, (iii) can they generate force and power twitching motility, (iv) are they involved in DNA uptake, (v) which genes are necessary for Tfp biogenesis and (vi) which genes are necessary for twitching motility? Answers to these questions are reported here.

## Results

### *S* *. sanguinis* surface‐associated motility is powered by the PilT retraction ATPase, which generates tremendous tension forces

**Figure 1:**
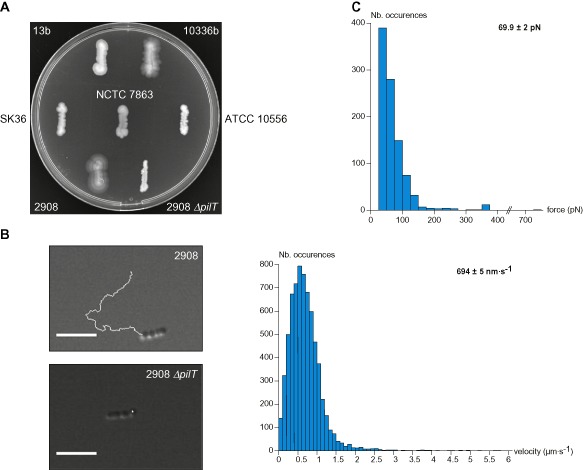
*S* *. sanguinis* exhibits PilT‐powered motility and exerts huge pulling forces, hallmarks of retractile Tfp. A. Macroscopic motility assay. Spreading zones, or lack thereof, around series of human isolates and a *Δpil* *T* mutant of 2908. B. Microscopic motility assay. Representative 30 s trajectories of movement of small chains of 2908 and *Δpil* *T* cells. Scale bar represents 5 μm. Corresponding movies are available as supplementary information (Movies S1 and S2). The histogram represents the distribution curve of velocities (in 100 nm s^−1^ intervals) measured for 2908 in three independent experiments. C. Measure of pulling forces exerted by 2908 using PoMPs force sensors. Movies showing the displacement of the micro‐pillar tips for WT and *Δpil* *T* are available as supplementary information (Movies S3 and S4). The histogram represents the distribution curve of pulling forces (in 25 pN intervals) measured in five independent experiments.

Studies by Henriksen *et al*. in the 1970s (Henriksen and Henrichsen, 1975; Henriksen and Eriksen, 1976) have shown that a form of motility consistent with twitching motility was a common property in *S. sanguinis*. This phenotype was readily assessed by the naked eye observation of thin spreading zones around bacteria grown on agar plates. We initially aimed at using the sequenced SK36 (Xu *et al*., 2007) for this study; however, it displayed no readily visible spreading zones (Fig. 1A). We therefore tested five *S. sanguinis* isolates shown long ago to be naturally competent and to exhibit a spreading phenotype on plates (Henriksen and Henrichsen, 1975). Spreading zones could readily be visualised for several of these strains, in particular 2908 (Fig. 1A), which was therefore chosen for further analyses.

In Gram‐negative bacteria that exhibit twitching motility, movement is mediated by Tfpa retraction, which is powered by the ATPase PilT (Skerker and Berg, 2001). We therefore created a *ΔpilT* mutant in strain 2908 (see below) and tested it using the above plate motility assay. As seen in Fig. 1A, in contrast to the wild‐type (WT) strain, *ΔpilT* exhibited no spreading zones. Motility was next assessed at the cellular level by tracking under the microscope the movement of small chains of cells attached to a glass coverslip (Fig. 1B). Short‐duration movies illustrating the movement of WT and *ΔpilT* are included in supplementary information (Movies S1 and S2). In contrast to *ΔpilT* that was only subjected to Brownian motion, WT showed ‘train‐like’ directional motion, with sometimes marked kinks. Motion proceeded mainly parallel to the long axis of bacterial chains with an instantaneous velocity of 694 ± 5 nm s^−1^ (mean ± standard error, *n* = 6,806) (Fig. 1B). This is the first molecular evidence that colony spreading, found long ago to be common in *S. sanguinis* isolates (Henriksen and Henrichsen, 1975), is powered by PilT and indeed corresponds to twitching motility.

Next, we aimed to measure the tension forces exerted by *S. sanguinis*, something that was done only for a couple of Gram‐negative Tfpa‐expressing species (Maier *et al*., 2002; Biais *et al*., 2008; Clausen *et al*., 2009). We used polyacrylamide micro‐pillars (PoMPs) as force sensors, as previously done for *Neisseria gonorrhoeae* (Biais *et al*., 2008). In brief, we recorded and analysed the displacement of micro‐pillar tips of calibrated stiffness upon bacterial attachment, which allows the corresponding tension forces exerted by the bacteria to be calculated. Movies of PoMPs assays with WT and *ΔpilT* are included in supplementary information (Movies S3 and S4). While no displacement of PoMPs was seen with *ΔpilT*, indicating that this is a PilT‐dependent process, WT exerted transient pulls on PoMPs of 69.9 ± 2 pN (mean ± standard error, *n* = 962) (Fig. 1C), with forces in excess of 700 pN being recorded. Taken together, these findings confirm that *S. sanguinis* motility is powered by the PilT retraction ATPase, which can generate hundreds of pN of tensile forces.

### A 22 kb cluster encodes all the proteins involved in Tfp biology in 2908

**Figure 2:**
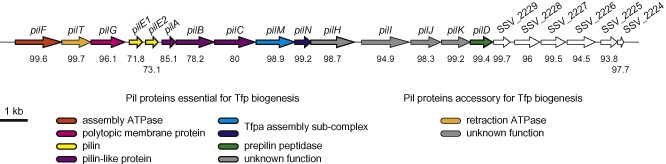
Genomic organisation of the *pil* locus in *S* *. sanguinis* 2908. All the genes are drawn to scale, with the scale bar representing 1 kb. Genes essential for Tfp biogenesis are boxed by a thick line. Genes encoding proteins predicted to play a role in Tfp biology based on the presence of signature sequence motifs have been colour coded and were named, when possible, after their homologues in *N* *. meningitidis*. Genes encoding proteins with no signature motifs but shown in this study to play a role in Tfp biology are in grey, whereas genes with (so far) no role in Tfp biology are in white. Values (in %) under each gene indicate the level of aa identity of the corresponding proteins in 21 other sequenced *S* *. sanguinis* genomes.

To facilitate our studies, we sequenced and annotated the genome of *S. sanguinis* 2908. The draft genome assembly is 2.3 Mb long with a G + C content of 43.3%, in line with SK36's complete genome (Xu *et al*., 2007). Comparative analysis revealed that the two genomes are essentially co‐linear, with similar coding densities and comparable numbers of genes (90% of which are orthologues). The 2908 genome annotation revealed that all the genes encoding distinctive proteins involved in Tfp biology (Table S1) cluster together in a locus hereafter named *pil* (Fig. 2). Synteny maps with other streptococcal genomes show that the *pil* locus is (i) 22 kb long, (ii) conserved in all *S. sanguinis* strains (except SK49) and (iii) absent in all other streptococcal species, none of which exhibits twitching motility. The G + C content of the *pil* locus is similar to the rest of the genome, arguing against recent horizontal acquisition. The *pil* locus in 2908 encompasses 21 genes, and most of the corresponding proteins are virtually identical in each sequenced *S. sanguinis* isolate (Fig. 2). The main difference is the presence, in half of these genomes, of three pilin genes rather than the two found in 2908. Proteins with distinctive Tfp biology sequence motifs (Table S1) were named after their homologues in *N. meningitidis* (PilD, PilE1, PilE2, PilF, PilG, PilM, PilN and PilT), one of the historic Tfpa model species (Pelicic, 2008). The other genes that were found in this study to play a role in Tfp biology (see below) were named in alphabetic order from left to right in Fig. 2 using the remaining letters (*pilA*, *pilB*, *pilC*, *pilH*, *pilI*, *pilJ* and *pilK)*. Unfortunately, this means that these genes are not homologous to genes with the same name in *N. meningitidis*. To avoid misunderstandings, we will when necessary label these proteins with a subscript to indicate the species they come from, *e.g.* PilH_SS_ or PilH_NM_ for *S. sanguinis* and *N. meningitidis* PilH proteins respectively.

Importantly, all the proteins that are universally conserved in Tff biogenesis are encoded in the *pil* locus (Table S1), *i.e.* two putative pilus subunits (PilE1 and PilE2), a prepilin peptidase (PilD), a traffic ATPase powering filament assembly (PilF) and a polytopic membrane protein of unclear function (PilG). In addition, the *pil* locus encodes several other proteins often involved in Tfp biology, the already mentioned PilT ATPase, PilM and PilN involved in Tfpa biogenesis (Carbonnelle *et al*., 2006) and three pilin‐like proteins (PilA_SS_, PilB_SS_ and PilC_SS_) with putative class III signal peptides (Fig. S1). Taken together, these findings suggest that the *pil* locus most likely encodes all the genes involved in Tfp biology in *S. sanguinis*. We have therefore engineered deletion mutants in each of these 21 genes, together with a double *ΔpilE1ΔpilE2* mutant in which both pilin genes were simultaneously deleted.

### pilE1 and pilE2 encode type IVa prepilins that are processed by the prepilin peptidase PilD

**Figure 3:**
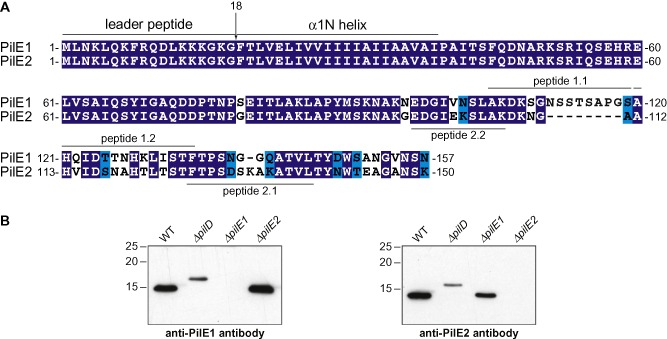
PilE1 and PilE2 are type IVa prepilins produced during growth *in vitro* and processed by the prepilin peptidase PilD. A. Sequence alignment of PilE1 and PilE2 prepilins encoded in 2908 genome. Residues were shaded in dark blue (identical), light blue (conserved) or unshaded (different). The class III signal peptide is highlighted, with the predicted processing site by the prepilin peptidase PilD indicated by a vertical arrow. Peptides (two per protein) used to generate antibodes are also indicated. B. Immunoblot analysis of PilE1 and PilE2 expression and processing by PilD. Whole‐cell protein extracts were probed using anti‐PilE1 or anti‐PilE2 antibodies. Protein extracts were quantified and equalised, and equivalent amounts of total proteins were loaded in each lane. Molecular weights are indicated in kDa.

The *pil* locus contains two genes, *pilE1* and *pilE2*, predicted to encode canonical type IV prepilins of 157 and 150 residues respectively. These two proteins display a high degree of sequence homology (Fig. 3A). Most of the diversity lies in their C‐terminal 30% as their first 110 residues are almost identical. Each protein harbours the same class III signal peptide (Fig. 3A), which is key both for processing by the prepilin peptidase PilD and subsequent polymerisation within filaments (Berry and Pelicic, 2015). PilE1 and PilE2 can be classified as type IVa prepilins because their N‐terminal hydrophobic stretch shares substantial sequence homology with other pilins of this sub‐class (Fig. S1).

PilE1 and PilE2 are predicted to be processed by the prepilin peptidase PilD after the Gly ending their leader peptide. This would lead to mature pilins of 14.7 and 14 kDa, respectively, 18 residues shorter than their 16.9 and 16.2 kDa precursors (Table S1). To start with the experimental characterisation of PilE1 and PilE2, we generated rabbit antisera against each protein. These antisera were used to confirm by immunoblotting that both proteins are expressed by *S. sanguinis* as they could be detected in whole‐cell protein extracts from WT, but not from *ΔpilE1* and *ΔpilE2* mutants (Fig. 3B). The absence of cross‐reaction indicates that each serum is specific for the protein against which it was raised. Importantly, PilE1 and PilE2 prepilins are both cleaved by PilD as confirmed by the detection of proteins of slightly higher molecular weight in *ΔpilD* (Fig. 3B), with masses consistent with those expected for unprocessed precursors.

### *S* *. sanguinis* 2908 produces Tfpa containing both PilE1 and PilE2

**Figure 4:**
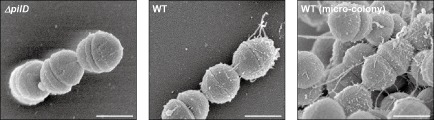
*S* *. sanguinis* 2908 produces Tfp. Piliation in the WT strain and *Δpil* *D* mutant was assessed by SEM. The scale bar represents 500 nm.

**Figure 5:**
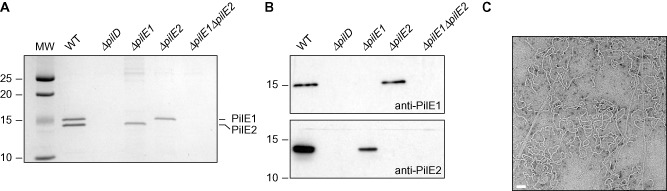
Purified *S* *. sanguinis* 2908 Tfp are composed of two pilins in comparable amounts. A. SDS‐PAGE/Coomassie analysis of purified *S* *. sanguinis* Tfp. Samples were prepared from cultures adjusted to the same OD_600_, separated by SDS‐PAGE and stained with Coomassie blue. Identical volumes were loaded in each lane. A molecular weight marker (MW) was run in the first lane. Molecular weights are indicated in kDa. B. Immunoblot analysis of pilus preparations using anti‐PilE1 or anti‐PilE2 antibodies. Molecular weights are indicated in kDa. C. Analysis of WT pilus preparations by TEM after negative staining. Scale bars represent 100 nm.

Although filaments with morphological features of Tfp were detected by transmission electron microscopy (TEM) on the surface of several *S. sanguinis* strains (Henriksen and Henrichsen, 1975; Gaustad and Froholm, 1984), molecular evidence that this species produces Tfp was missing. We therefore used scanning electron microscopy (SEM) to compare WT and *ΔpilD*, which is expected to be non‐piliated. While *ΔpilD* displayed a completely smooth surface, many rope‐like filaments of various lengths, likely to be bundles of Tfp, could be seen on the surface of the WT strain (Fig. 4). Inside microcolonies, these filaments form a dense network of connections between cells. Next, we designed a reliable pilus purification procedure, robust enough to determine *S. sanguinis* pilus composition and analyse piliation in each of the mutants we constructed. As assessed by Coomassie staining after SDS‐PAGE (Fig. 5A), this method yielded fairly pure pilus preparations consisting mainly of two bands, the size of which was consistent with mature PilE1 and PilE2 (14.7 and 14 kDa respectively). Importantly, these bands were absent in preparations obtained from *ΔpilD*, confirming that this mutant is non‐piliated. We confirmed by mass spectrometry (data not shown) and immunoblotting (Fig. 5B) that the upper band corresponds to PilE1, whereas the lower one corresponds to PilE2. Confirmation that WT purified fractions actually consist of filaments was obtained by TEM, which showed an abundance of pili displaying (for an unknown reason) two different morphologies (Fig. 5C). The overwhelming majority of these filaments were approx. 12 nm thick and wavy, whereas some had a somewhat more classical Tfp morphology, *i.e.* they were straight, thinner (approx. 6 nm wide) and sometimes formed small bundles.

Together, the above findings are the first molecular evidence that filaments found long ago to be common in *S. sanguinis* isolates are Tfpa. Intriguingly, as assessed by Coomassie staining (Fig. 5A), PilE1 and PilE2 were present in comparable amounts in Tfp purified from WT. This is highly unusual as Tfp are composed of one major subunit and, sometimes, one or several minor (low abundance) ones (Berry and Pelicic, 2015). Often, mutants in the major pilin are non‐piliated, whereas those in minor pilins still express pili (Brown *et al*., 2010). We therefore tested whether one of the two pilins in 2908 was more important for piliation by performing pilus preparations on *ΔpilE1* and *ΔpilE2*. Strikingly, each mutant yielded preparations consisting of a single band (Fig. 5A), corresponding to the remaining pilin as shown by immunoblotting (Fig. 5B). Very often, but not always, these preparations were less concentrated than those made from WT, suggesting that the mutants produced less pili (data not shown). As seen by TEM, the *ΔpilE1* and *ΔpilE2* filaments had a morphology similar to WTs, with a mixture of straight/thin and wavy/thick filaments showing that the different morphologies do not correlate with different pilus compositions (data not shown). These findings indicate that none of the two pilins in *S. sanguinis* 2908 is absolutely required for piliation. However, at least one of these subunits must be expressed for Tfp to be produced as demonstrated by the absence of bands in pilus preparations from a double *ΔpilE1ΔpilE2* mutant (Fig. 5A).

Interestingly, filaments purified from some other *S. sanguinis* isolates that we analysed for twitching motility also contained more than one pilin (Fig. S2). This is therefore a common property of *S. sanguinis* Tfp and is consistent with the presence of multiple *pilE* genes in all the currently available genomes. In contrast, and for an unknown reason, no filaments could be purified from SK36, which might explain why this strain did not exhibit detectable twitching motility.

### Tfp biogenesis in *S* *. sanguinis* requires only 10 proteins

**Figure 6:**
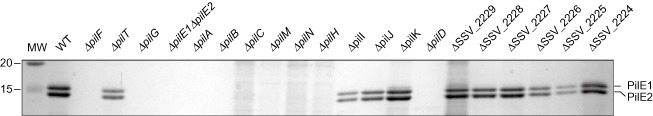
Ten proteins are essential for Tfp biogenesis in *S* *. sanguinis*. Pilus preparations made from mutants in each gene in the *pil* locus were separated by SDS‐PAGE and stained with Coomassie blue. The WT strain was included as a control. Samples were prepared from cultures adjusted to the same OD_600_.

Large‐scale genetic analyses defining the complete set of proteins involved in Tfp biogenesis have previously been done only in a handful of Gram‐negative species (Pelicic, 2008). Therefore, because only a subset of the 15 Pil proteins usually conserved in Tfpa‐expressing bacteria (Pelicic, 2008; Berry and Pelicic, 2015) are found in *S. sanguinis* and some proteins encoded by the *pil* locus are specific to this species (Table S1), the next question we asked was how many genes in this locus are essential for pilus biogenesis. We therefore systematically made pilus preparations from each of the single and/or double mutants we have generated and analysed these by SDS‐PAGE and Coomassie staining (Fig. 6). Out of the 20 mutants that were analysed, 10 yielded preparations composed of PilE1 and PilE2 just like the WT strain and are therefore piliated. In contrast, 10 mutants were non‐piliated as no pilin bands were present in the corresponding pilus preparations (Fig. 6). Therefore, 10 proteins only are essential for Tfpa biogenesis in *S. sanguinis*, indicating that the corresponding machinery is significantly simpler than in traditional Gram‐negative Tfpa models.

### Four Pil proteins dispensable for piliation are key for surface‐associated motility

**Figure 7:**
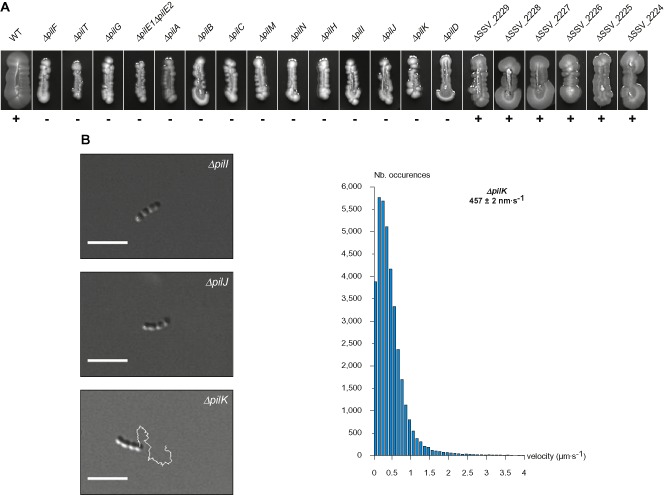
Four proteins dispensable for piliation modulate twitching motility. A. Macroscopic motility assay. Mutants in each gene in the *pil* locus were analysed for their ability to produce spreading zones on plates. WT strain was included as a control. B. Microscopic motility assay for *Δpil* *I*, *Δpil* *J* and *Δpil* *K* piliated mutants. Representative 30 s trajectories of movement of small chains of cells are shown. Corresponding movie for *Δpil* *K* is available as supplementary information (Movie S5).The histogram represents the distribution curve of velocities (in 100 nm s^−1^ intervals) measured for *Δpil* *K* in three independent experiments.

Using our plate motility assay, we next determined which genes in the *pil* locus are essential for *S. sanguinis* motility. Out of the 20 mutants that were analysed, 14 lacked spreading zones and are therefore non‐motile, while six were apparently not affected (Fig. 7A). As could be expected, all the non‐piliated mutants were non‐motile. Critically, in addition to the already analysed *ΔpilT*, three other mutants that still produce Tfp were also non‐motile. They harbour mutations in *pilI*, *pilJ* and *pilK*, which are not found in Gram‐negative species. Strikingly, these mutants exhibited different motility phenotypes when assessed at the cellular level. While *ΔpilI* and *ΔpilJ* showed no cellular movement similarly to *ΔpilT*, *ΔpilK* was motile (Fig. 7B). However, *ΔpilK* cellular motility was different from WT's. A movie illustrating the movement of *ΔpilK* is included in supplementary information (Movie S5). *ΔpilK* crawled ‘crabwise’ perpendicular to the long axis of the bacterial chains and frequently reversed gear. The instantaneous velocity of this mutant was roughly two‐thirds of WT's at 457 ± 2 nm s^−1^ (mean ± standard error, *n* = 36,508) (Fig. 7B). These findings suggest that, together with PilT that is the pilus retraction motor, PilI, PilJ and PilK play important, but different, roles in motility in *S. sanguinis*.

### Tfp are not involved in DNA transformation in *S* *. sanguinis*

**Figure 8:**
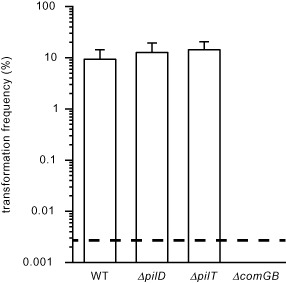
*S* *. sanguinis* Tfp are not involved in natural competence. Competence for DNA transformation was quantified using a PCR fragment conferring resistance to streptomycin. In *Δcom* *GB*, we deleted a component of the (pseudo)pilus Tff nano‐machine involved in DNA uptake in other naturally competent species. Results are expressed as transformation frequencies, *i.e.* number of Str^R^ CFU relative to total number of CFU, and are the mean ± standard deviation of at least four independent experiments. The dotted line indicates the lower limit of detection.

Finally, as competence is Tfp‐dependent in some naturally transformable species, we assessed whether this was also the case in *S. sanguinis*. Strain 2908 was found to be highly competent as 9.34 ± 5% of the cells (mean ± standard deviation, *n* = 6) could readily be transformed (Fig. 8). Similar transformation frequencies were measured for *ΔpilT* or *ΔpilD* harbouring non‐retractable Tfp or no Tfp at all respectively. These results show that competence for DNA transformation in *S. sanguinis* is Tfp independent. Closer inspection of 2908 genome revealed that all the genes encoding a competence (pseudo)pilus, a Tff nano‐machine involved in DNA uptake in naturally competent Gram‐positive species (Chen and Dubnau, 2004; Laurenceau *et al*., 2013), were present (Fig. S3). When one of these genes (*comGB*) was deleted, competence was completely abolished (Fig. 8). Together, these findings show that Tfp are not involved in DNA uptake in *S. sanguinis*, which is instead mediated by a distinct Tff nano‐machine.

## Discussion

Owing to their near ubiquity in prokaryotes (Berry and Pelicic, 2015), a better understanding of Tff biology is highly desirable. Much of our current understanding comes from study of Tfp in a few related Gram‐negative models. This has often allowed the results obtained in one species to be confirmed in others, demonstrating the universality of some of the molecular steps involved. However, important discrepancies have also been reported, not the least of which might be different filament assembly machineries proposed in *N. meningitidis* and *Pseudomonas aeruginosa* (Carbonnelle *et al*., 2006; Takhar *et al*., 2013). It has been predicted that the study of Tfp in a phylogenetically distant Gram‐positive species has the potential to shine new light on poorly understood steps and maybe settle some of the existing disputes (Melville and Craig, 2013; Berry and Pelicic, 2015). However, this has been so far hindered by the lack of a genetically tractable piliated monoderm species. In this report, we have addressed this limitation and the notable findings listed below firmly establish *S. sanguinis* as a new model species for studying Tfpa.

The first important finding in this study is that *S. sanguinis* expresses *bona fide* Tfpa. Indeed, we have shown that strain 2908 filaments (i) are composed of type IVa pilins, (ii) are synthesized by a machinery containing some proteins found only in Tfpa‐expressing species (Berry and Pelicic, 2015), (iii) can generate huge tension forces and (iv) power intense surface‐associated motility. Tension forces measured for *S. sanguinis* are remarkably similar to those reported in Gram‐negative Tfpa‐expressing species (Maier *et al*., 2002; Biais *et al*., 2008; Clausen *et al*., 2009), which is perhaps unexpected considering that these bacteria have dramatically different cell wall architectures. However, *S. sanguinis* filaments also display several important differences with prototypical Gram‐negative Tfpa, which are as informative as the above similarities. Perhaps the most important difference is that *S. sanguinis* filaments are composed of two pilins in comparable amounts, which goes against the notion that Tfp are always polymers of one subunit. Although the possibility that two distinct homo‐polymers coexist cannot be excluded, it is likely that *S. sanguinis* pili are hetero‐polymers considering the identical N‐termini of PilE1 and PilE2. Unfortunately, we could not confirm this by immuno‐TEM because our antibodies do not work well in this assay (data not shown). The second notable difference is the size of PilE1 and PilE2 leader peptide (18 aa), which is much longer than in canonical type IVa pilins, and the genomic organisation of the *pil* genes, which are clustered in *S. sanguinis* while they are scattered throughout the genome in Gram‐negative Tfpa‐expressing species (Pelicic, 2008). These findings are (misleadingly) more in line with model Tfpb‐expressing species, showing that some of the traditional parameters used to define Tfpa and Tfpb are not valid anymore.

The most significant achievement in this study is the systematic genetic analysis of the *pil* locus in *S. sanguinis*, which has never been performed for a non‐Proteobacterium before. This revealed that 15/21 genes in this locus play a role in Tfp biogenesis and/or Tfp‐mediated motility. It is possible that the remaining six genes also play a role in Tfp biology by modulating other Tfp‐mediated properties, which remains to be assessed in future studies. However, Tfp are clearly dispensable for natural transformation in *S. sanguinis* where DNA uptake is mediated by a competence (pseudo)pilus like in other competent Gram‐positive species (Chen and Dubnau, 2004; Laurenceau *et al*., 2013). This makes *S. sanguinis* the first Gram‐positive species with two distinct, independent Tff nano‐machines.

The finding that 10 proteins only are necessary for piliation in *S. sanguinis* makes it the simplest model bacterial species for studying Tfp biogenesis, on a par with Tfpb‐expressing *Vibrio cholerae* (Pelicic, 2008). Interestingly, these 10 proteins are also found in Clostridia (Fig. S4), another class of Tfpa‐expressing Firmicutes (Melville and Craig, 2013), where they are expected to play similar roles. Comparison of these proteins with the 15 necessary for pilus biogenesis in *N. meningitidis*, which are shared by 270 species of Tfpa‐expressing Proteobacteria (Berry and Pelicic, 2015), offers novel insight in Tfp biogenesis. It reveals that while six components are common to the two systems (PilD, PilE, PilF, PilG, PilM and PilN) and there are three putative pilin‐like proteins in *S. sanguinis* (PilA_SS_, PilB_SS_ and PilC_SS_) instead of four in *N. meningitidis* (PilH_NM_, PilI_NM_, PilJ_NM_ and PilK_NM_), five *N. meningitidis* proteins (PilC_NM_, PilO, PilP, PilQ and PilW) are absent in *S. sanguinis*, whereas only one is specific to *S. sanguinis* (PilH_SS_). As the four pilin‐like proteins conserved in Gram‐negative species that are thought to prime filament assembly (Cisneros *et al*., 2012) are absent in *S. sanguinis*, this process is likely to be radically different in Gram‐positive bacteria. Indeed, the three *S. sanguinis* pilin‐like proteins are unlikely to play such a fundamental role since (together with the pilins PilE1 and PilE2) they are far more variable (70–80% aa identity in different isolates) than the rest of the Pil proteins (95–99% aa identity) (Fig. 2). This observation is more consistent with a pilus localisation and exposure to the host immune system driving variability, suggesting that PilA_SS_, PilB_SS_ and PilC_SS_ may be minor pilus subunits. The identity of some of the five *N. meningitidis* proteins absent in *S. sanguinis* is also thought provoking and has implication for our understanding of pilus assembly. As mentioned, there are two different models for pilus assembly suggesting either that a PilMNOP sub‐complex is involved (Carbonnelle *et al*., 2006; Georgiadou *et al*., 2012), or that filaments are assembled by PilG (Takhar *et al*., 2013) while PilMNOP acts as a ‘connecting module’ between inner and outer membrane sub‐complexes (Ayers *et al*., 2010; Nivaskumar and Francetic, 2014). The mere presence of PilM and PilN in *S. sanguinis* and their involvement in Tfp biogenesis is a strong evidence that they are not a ‘connecting module’ as there is no outer membrane in this species. However, the absence of PilO and PilP in *S. sanguinis* is also a clear indication that Tfpa assembly can occur in their absence, at least in a Gram‐positive background. While as discussed elsewhere (Berry and Pelicic, 2015), PilP might have been attributed a role in pilus assembly merely because it is essential for stability of the PilN‐PilO hetero‐dimer (Georgiadou *et al*., 2012), the absence of PilO in *S. sanguinis* is more puzzling. It is possible, however, considering the similar 3D structures of PilN and PilO (Sampaleanu *et al*., 2009; Karuppiah *et al*., 2013), that *S. sanguinis* uses a PilN‐PilN homo‐dimer in place of the PilN‐PilO hetero‐dimer found in Gram‐negative species. This hypothesis is in line with the apparent simplicity of the Gram‐positive Tfp biogenesis machinery.

The finding that biophysical parameters of force generation in *S. sanguinis* are remarkably similar to those in Gram‐negative Tfpa‐expressing species offers the unprecedented possibility to characterise pilus retraction/force generation/motility in the absence of an outer membrane. We have identified a subset of proteins dispensable for pilus biogenesis but required for optimal motility. Unlike PilT, the other proteins (PilI, PilJ and PilK) are not found in Gram‐negative Tfpa‐expressing species. Because the phenotypic defects in the corresponding mutants are indistinguishable from *ΔpilT*'s, it is possible that PilI and PilJ are involved in facilitating pilus retraction across the very thick layer of PG in Gram‐positive species. In contrast, the intriguing finding that *ΔpilK* is incapable of macroscopic motility but is motile at the cellular level is a clear indication that PilK plays a different role. As the directionality of motion seems affected in *ΔpilK* when compared with WT, PilK's role might be to ‘steer the trains of cells’ to ensure that net macroscopic movement occurs. This will be the topic of future experiments.

In conclusion, this study provides the first global view of the multi‐protein machinery at play in Tfp biology in a non‐Proteobacterium species, making the Gram‐positive *S. sanguinis* a new Tfp model species in its own right. This species genetic tractability and the observations listed above pave the way for further investigations, which are expected in the years to come to improve our fragmentary understanding of a fascinating and widespread class of prokaryotic filamentous nano‐machines.

## Experimental procedures

### Strains and growth conditions

The strains of *S. sanguinis* used in this study are listed in Table S2. Bacteria were grown on plates containing Todd Hewitt (TH) medium (Difco) and 1% agar (Difco). Plates were incubated overnight (O/N) at 37°C in a 3.5 l anaerobic jar (Oxoid), under anaerobic conditions generated using Anaerogen sachets (Oxoid). Liquid cultures were grown statically under aerobic conditions in THT, *i.e.* TH broth containing 0.05% tween 80 (Merck) to limit bacterial clumping. When necessary, 500 μg ml^−1^ kanamycin or 100 μg ml^−1^ streptomycin (both from Sigma) was used for antibiotic selection.

Using a splicing PCR strategy that has been previously shown to generate non‐polar mutants (Xu *et al*., 2011; Georgiadou *et al*., 2012), we have constructed a series of mutants in *S. sanguinis* 2908. High‐quality genomic DNA was prepared from O/N liquid cultures using the XIT Genomic DNA from Gram‐Positive Bacteria kit, as instructed by the manufacturer (G‐Biosciences). For each target gene, 500–800 bp fragments upstream (Up) and downstream (Down) were amplified using F1/R1 and F2/R2 pairs of primers (Table S3). R1 and F2 were designed to cleanly delete target genes from their start codon to approx. 30 bp before the stop codon (in order to preserve putative ribosomal binding sites that might be used by genes immediately downstream). For *pilH*, the start of which overlaps with the end of upstream *pilN* gene, the deletion was from the seventh residue. In addition, these primers contained 23‐mer overhangs complementary to the aph1 and aph2 primers used to amplify a 795 bp promoterless *aphA‐3* gene (Aph), which encodes an aminoglycoside phosphotransferase conferring resistance to kanamycin (Trieu‐Cuot and Courvalin, 1983) (Km^R^). Equal volumes of the three PCR fragments (Up, Down and Aph) were combined and spliced together by PCR. All PCRs were done using high‐fidelity Herculase II Fusion DNA Polymerase (Agilent). Spliced PCR fragments (5 μl) were directly transformed into strain 2908 (see below), which is naturally competent. Allelic exchange mutants were selected by plating on kanamycin‐containing TH agar plates and growing O/N under anaerobic conditions. Genomic DNA was extracted from at least two Km^R^ transformants, and it was tested by PCR and sequenced using the corresponding F1 and R2 primers, in order to confirm that the desired allelic exchange had taken place.

### Sequence and annotation of *S* *. sanguinis* 2908 genome

Strain 2908 genome was sequenced on an Illumina MiSeq platform. Paired‐end 150 bp reads were generated from a library of 416 bp average size. A total of 245 Mb of data was obtained, representing an approx. 100‐fold genome coverage. The reads were assembled into 17 contigs (N50 of 558,829 bp) using an iterative sequence assembly process defined previously (Chewapreecha *et al*., 2014). Contigs were manually ordered using the Artemis Comparison Tool, relatively to *S. sanguinis* SK36 published genome (Xu *et al*., 2007), and placed into a single scaffold. Raw sequence data for strain 2908 was deposited into the European Nucleotide Archive under the accession number ERR245866. Annotation was performed using the MicroScope annotation pipeline and its MaGe web interface (Vallenet *et al*., 2013), as described elsewhere (Rusniok *et al*., 2009). In brief, coding sequences (CDS) were assigned a unique SSV_ identifier and were submitted to automatic functional annotation in MicroScope. Functional annotation and syntactic homogeneity of each CDS were then refined manually during iterative rounds of inspection. The annotated draft genome of strain 2908 was deposited into the European Nucleotide Archive under the accession number PRJEB7884. To facilitate genomic comparisons, the published SK36 genome and 20 other publicly available draft *S. sanguinis* genomes (ATCC 29667, SK1, SK1056, SK1057, SK1058, SK1059, SK1087, SK115, SK150, SK160, SK330, SK340, SK353, SK355, SK405, SK408, SK49, SK678, SK72, VMC66) have also been automatically (re)annotated. These datasets are stored in a publicly accessible database in MicroScope, which we named SanguiniScope. All the genomic analyses have been performed using embedded softwares in MicroScope.

### SDS‐PAGE, antisera and immunoblotting

Whole‐cell protein extracts were prepared as follows. Liquid cultures were grown O/N in THT. The next day, these cultures were used to re‐inoculate 100 ml of THT and grown statically until the OD_600_ reached 0.6–1, at which point ODs were normalised if necessary. Bacteria were pelleted at 4°C by centrifuging for 15 min at 14,000 *g*. Pellets were re‐suspended in 1 ml of phosphate buffer saline (PBS) and transferred to Lysing Matrix B 2 ml tubes containing 0.1 mm silica beads (MP Biomedicals). Cells were disrupted mechanically using a FastPrep‐24 homogeniser (MP Biomedicals) set at 6.5 speed, during five cycles of 1 min homogenisation/1 min rest. Cell debris and beads were then pelleted by a brief centrifugation, and 900 μl of supernatant was transferred to a new tube. Protein concentration was determined using the Bio‐Rad Protein Assay, as instructed by the manufacturer. Separation of the proteins by SDS‐PAGE and subsequent blotting to Amersham Hybond ECL membranes (GE Healthcare) was carried out using standard molecular biology techniques. For immunoblotting, blocking of membranes, incubation with primary antibody (1/2,000 dilution) and/or secondary ECL HRP‐linked anti‐rabbit antibody (GE Healthcare) (1/10,000 dilution), and detection using Amersham ECL Prime (GE Healthcare) was done following the manufacturer's instructions. Antisera against PilE1 and PilE2 were produced by Eurogentec by immunising rabbits with a mixture of two different peptides. Peptides corresponding to residues 105–119 and 120–134 of PilE1, and 97–106 and 126–138 of PilE2 were used for the immunisations (see Fig. 3A). These peptides were used to affinity‐purify the antibodies.

### Tfp visualisation and purification

Presence of Tfp on bacteria was assessed by SEM as follows. Round cover slips (18 mm diameter) were added at the bottom of the wells of a 12 well tissue culture plate. One ml of THT medium was added to each well, before inoculating with 10 μl of liquid cultures (OD_500_ of 0.5) and incubating 3 h at 37°C in a humidified atmosphere with 5% CO_2_. Cells were then fixed for 1 h at room temperature by the addition of 1 ml of 4% glutaraldehyde (in PBS). Samples were washed three times with PBS and then gradually dehydrated by passages through a series of 50, 70, 80, 90 and 100% ethanol solutions (5 min for each step). Finally, samples were critical point dried and sputtered with 10 nm gold palladium before being imaged on an Hitachi S4700 Scanning Electron Microscope operated at 3 kV.

Tfp of *S. sanguinis* 2908 were purified as follows. Liquid cultures were grown O/N in THT. The next day, these cultures were used to re‐inoculate 100 ml of THT and grown statically until the OD_600_ reached 1–1.5, at which point ODs were normalised if necessary. Bacteria were pelleted at 4°C by centrifugation for 10 min at 6,000 *g* and pellets were re‐suspended in 1 ml (20 mM Tris pH7.5, 50 mM NaCl). This suspension was vortexed for 2 min at full speed to shear Tfp, before bacteria were pelleted again as above. Nine hundred microlitres of supernatant was then transferred to a new tube, and the pili were pelleted at 4°C by ultra‐centrifugation for 1 h at 100,000 *g*. Pellets were resuspended in 40 μl (20 mM Tris pH7.5, 50 mM NaCl), separated by SDS‐PAGE and gels were stained using Bio‐Safe Coomassie stain (Bio‐Rad). Mass spectrometry identification of protein bands has been performed at the Taplin Mass Spectrometry Facility (Harvard Medical School). Purified filaments were visualised by TEM after negative staining using a Philips CM‐200 FEG microscope operated at 200 kV. In brief, 2 μl of purified Tfp was adsorbed for 1 min to a carbon‐coated 300 mesh copper grids (TAAB Laboratories Equipment). The filaments were then stained for 1 min with 2% phosphotungstic acid. Stain solution was gently drained off the grids, which were air‐dried before visualisation.

### Transformation of *S* *. sanguinis*

Strain 2908 was mainly transformed as described elsewhere (Paik *et al*., 2005). In brief, bacteria grown O/N in THT were back‐diluted 1/200 in THTS [THT supplemented with 2.5% heat‐inactivated horse serum (Sigma)] and incubated at 37°C until the OD_600_ reached 0.06–0.08. Competence was then induced using 500 ng ml^−1^ synthetic DLRGVPNPWGWIFGR (Håvarstein *et al*., 1997; Zhu *et al*., 2011) CSP (Peptide Protein Research) before transforming DNA was added to a 330 μl aliquot of competent cells. After incubation for 1 h at 37°C, the mixture was plated on suitable selective agar plates, which were grown O/N under anaerobic conditions.

For quantifying competence, we first selected a mutant of 2908 spontaneously resistant to streptomycin (Str^R^), which was found to harbour a mutation in the *rpsL* gene leading to a K_56_R change in the corresponding protein. A 2,184 bp PCR fragment encompassing the mutant *rpsL* was amplified using dedicated primers (Table S3), purified using QIAquick PCR purification kit (Qiagen) and used to transform the bacteria as above with minor modifications. Bacteria grown O/N were back‐diluted 1/13,000 in THS and incubated 1 h at 37°C. A 330 μl aliquot was then transferred to pre‐warmed tubes containing 70 ng CSP and 100 ng of purified *rpsL* PCR fragment. After 1 h at 37°C, the mixture was bath‐sonicated for 15 s using a S‐4000 sonicator (Qsonica) set at 10% amplitude to break chains of bacteria before making serial dilutions. Suitable dilutions were plated on non‐selective plates for counting total colony‐forming units (cfu) and on plates with streptomycin for counting transformants.

### Twitching motility assays

Twitching motility was assessed macroscopically on agar plates as follows. Bacteria grown O/N were streaked as straight lines on freshly poured TH plates containing 1% agar (Eiken Chemicals), which were incubated under anaerobic conditions. To provide the humid conditions necessary for twitching, which manifests itself a spreading zone around the streak line, the jar contained 25 ml water at the bottom. Movement was analysed microscopically as follows. Bacteria grown O/N on agar plates were resuspended in TH by vortexing for 2 min and adjusted to OD_500_ = 0.5. One millilitre of a 1,000‐fold dilution in THT was then added into an open experimental chamber with a glass bottom, which was incubated for 2 h at 37°C in a humidified atmosphere containing 5% CO_2_. The chamber was transferred to an upright Ti Eclipse microscope (Nikon) with an environment chamber maintaining 5% CO_2_ and 37°C throughout the experiment. Movies (10 Hz) of the motion of small bacterial chains were obtained and analysed offline in ImageJ, using a home‐written cross‐correlation algorithm. Cell speed was measured from the collected trajectories using Matlab.

Forces exerted by retracting *S. sanguinis* Tfp were measured using PoMPs as previously reported (Biais *et al*., 2008). Briefly, a silicon mold of micro‐pillars was inverted on a droplet of a polyacrylamide‐bisacrylamide mixture on an activated coverglass. The removal of the mold after reticulation of the gel enabled the obtention of a regular array of flexible micro‐pillars with a spring constant of 240 ± 30 pN μm^−1^. PoMPs were then coated for 1 h at 37°C with a 30 μg ml^−1^ solution of polylysine (in PBS) activated with SulfoSANPAH cross‐linker (Invitrogen). After three washes in water, PoMPs were coated for 1 h with 1/10 solution (Molecular Probes) of 20 nm carboxylated beads in water. PoMPs are then washed twice in water and once in PBS. One millilitre of a 1,000‐fold dilution in TH of a culture at OD_500_ = 0.5 was then added onto the PoMPs, the cover glass serving as the bottom of the experimental chamber, and incubated for 2 h at 37°C in a humidified atmosphere containing 5% CO_2_. As above, the PoMPs chamber was then transferred to the upright Ti Eclipse microscope, movies (10 Hz) were obtained and the deflection of the top of the PoMPs over time induced by attached bacteria was analysed offline in ImageJ, using a home‐written cross‐correlation algorithm. The forces exerted on the PoMPs were calculated from those deflections and the calibration of the pillars using Matlab.

## Supporting information

Supporting information
