# Supplementary material for: Functional analysis of an unusual type IV pilus in the Gram‐positive Streptococcus sanguinis
Source: Mol Microbiol. 2015 Oct 27;99(2):380–92. doi: 10.1111/mmi.13237 (PMC4832360; doi:10.1111/mmi.13237)
Supplement: Supplementary file 1 — Supporting information [file MMI-99-380-s001.zip › MMI_13237_supp-0006-Supplementary_material.pdf]

Supplementary Information

Functional analysis of an unusual type IV pilus in the Gram-positive *Streptococcus sanguinis*

Ishwori Gurung[1], Ingrid Spielman[2], Mark R. Davies[3,4], Rajan Lala[2], Peter Gaustad[5], Nicolas Biais[2] and Vladimir Pelicic[1,*]

[1]MRC Centre for Molecular Bacteriology and Infection, Imperial College London, London, United Kingdom

[2]Department of Biology, Brooklyn College of the City University of New York, New York, USA

[3]The Wellcome Trust Sanger Institute, Hinxton, Cambridge, United Kingdom

[4]Australian Infectious Diseases Research Centre, The University of Queensland, Brisbane, Queensland, Australia

[5]Department of Microbiology, Oslo University Hospital, Oslo, Norway

*For correspondence. E. mail: v.pelicic@imperial.ac.uk

Table S1. Proteins encoded by the *pil* locus of *S. sanguinis* 2908.

SSV_ gene	protein name	MW (kDa)	pI	predicted localization	InterPro domain(s)	(predicted) function
2244	PilF	62.2	5.19	cytoplasm	IPR001482	Tfp biogenesis motor
2243	PilT	39.3	6.60	cytoplasm	IPR006321	Tfp retraction motor
2242	PilG	44.9	8.77	membrane	IPR003004	Tfp biogenesis
2241	PilE1	16.9	9.36	pilus	IPR012902	pilin subunit
2240	PilE2	16.2	9.43	pilus	IPR012902	pilin subunit
2239	PilA	18	6.09	-	-	putative pilin-like protein
2238	PilB	51.4	9.15	pilus	IPR012902 & IPR002035	pilin-like protein
2237	PilC	54	9.20	pilus	IPR012902	pilin-like protein
2236	PilM	52.7	5.28	cytoplasm	IPR005883	Tfp biogenesis
2235	PilN	22.3	5.80	membrane	IPR007813	Tfp biogenesis
2234	PilH	56.6	5.47	-	-	Tfp biogenesis
2233	PilI	63.4	4.53	membrane	-	Tfp-mediated motility
2232	PilJ	39.3	4.23	cytoplasm	IPR011033	Tfp-mediated motility
2231	PilK	39.5	5.47	membrane	-	Tfp-mediated motility
2230	PilD	27.5	8.47	membrane	IPR000045 & IPR010627	prepilin peptidase
2229	-	21.5	9.64	membrane	IPR002901	glucosaminidase
2228	-	33.8	8.95	membrane	-	-
2227	-	34	8.37	membrane	-	-
2226	-	37.4	6.05	-	IPR027065	protease
2225	-	21.8	9.65	membrane	IPR024257	-
2224	-	8.6	8.35	cytoplasm	IPR010982	transcriptional regulator

Localization was predicted using PSORTb v3.0.2. Signature motifs in the different proteins were identified by scanning the InterPro 49.0 database.

Table S2. ***S. sanguinis*** **strains used in this study.**

name	genotype	description	source or reference
**wild-type strains**			
SK36		dental plaque isolate	(Xu *et al.*, 2007)
NCTC 7863		subacute endocarditis isolate	
ATCC 10556		subacute endocarditis isolate	
13b		throat swab isolate	(Henriksen & Eriksen, 1976)
2908		throat swab isolate	(Henriksen & Henrichsen, 1975)
**2908 mutants**			
2908-Str^R	$rpsL_{A167G}$	spontaneous Str^R mutant	this study
ΔpilF	ΔSSV_2244::*aphA-3*	*pilF* deletion mutant	this study
ΔpilT	ΔSSV_2243::*aphA-3*	*pilT* deletion mutant	this study
ΔpilG	ΔSSV_2242::*aphA-3*	*pilG* deletion mutant	this study
ΔpilE1	ΔSSV_2241::*aphA-3*	*pilE1* deletion mutant	this study
ΔpilE2	ΔSSV_2240::*aphA-3*	*pilE2* deletion mutant	this study
ΔpilE1ΔpilE2	ΔSSV_2241SSV_2240::*aphA-3*	double *pilE1pilE2* deletion mutant	this study
ΔpilA	ΔSSV_2239::*aphA-3*	*pilA* deletion mutant	this study
ΔpilB	ΔSSV_2238::*aphA-3*	*pilB* deletion mutant	this study
ΔpilC	ΔSSV_2237::*aphA-3*	*pilC* deletion mutant	this study
ΔpilM	ΔSSV_2236::*aphA-3*	*pilM* deletion mutant	this study
ΔpilN	ΔSSV_2236::*aphA-3*	*pilN* deletion mutant	this study

ΔpilH	ΔSSV_2234::*aphA-3*	*pilH* deletion mutant	this study
ΔpilI	ΔSSV_2233::*aphA-3*	*pilI* deletion mutant	this study
ΔpilJ	ΔSSV_2232::*aphA-3*	*pilJ* deletion mutant	this study
ΔpilK	ΔSSV_2231::*aphA-3*	*pilK* deletion mutant	this study
ΔpilD	ΔSSV_2230::*aphA-3*	*pilD* deletion mutant	this study
ΔSSV_2229	ΔSSV_2229::*aphA-3*	SSV_2229 deletion mutant	this study
ΔSSV_2228	ΔSSV_2228::*aphA-3*	SSV_2228 deletion mutant	this study
ΔSSV_2227	ΔSSV_2227::*aphA-3*	SSV_2227 deletion mutant	this study
ΔSSV_2226	ΔSSV_2226::*aphA-3*	SSV_2226 deletion mutant	this study
ΔSSV_2225	ΔSSV_2225::*aphA-3*	SSV_2225 deletion mutant	this study
ΔSSV_2224	ΔSSV_2224::*aphA-3*	SSV_2224 deletion mutant	this study
ΔcomGB	ΔSSV_0098::*aphA-3*	*comGB* deletion mutant	this study

Table S3. Primers used in this study.

name	sequence
aph1	ATGGCTAAAATGAGAATATCACC
aph2	CTAAAACAATTCATCCAGTAAAA
pilF-F1	GAGAAAGCGACAAGGAGGTG
pilF-R1	GGTGATATTCTCATTTTAGCCATTTGTTTCTCCTGTCTGTGATTTT
pilF-F2	TTTTACTGGATGAATTGTTTTAGCAACGATTGAAGAAGCAATGA
pilF-R2	TTCCTTATCCCGCATCTCAC
pilT-F1	GAAGGGGTCAACCAAGTTCA
pilT-R1	GGTGATATTCTCATTTTAGCCATCCTAAACTTCCCCTTCTAGACT
pilT-F2	TTTTACTGGATGAATTGTTTTAGAAGTCGAAAAAGCCCTAGGAA
pilT-R2	AGGCCAAGACTGCTCCTACA
pilG-F1	AACAGGAAGCGGAAAGTCAA
pilG-R1	GGTGATATTCTCATTTTAGCCATGCTCTAGTACCCCACTATTCC
pilG-F2	TTTTACTGGATGAATTGTTTTAGCTCTGTCATGCTGCCAATGT
pilG-R2	AAACAGTAGCTTGGCCTCCA
pilE1-F1	GGGGATTCTGGTCTTCCTCT
pilE1-R1	GGTGATATTCTCATTTTAGCCATTTTGAATAGATCTCCTGTTTTT
pilE1-F2	TTTTACTGGATGAATTGTTTTAGCGACTGGTCTGCTAATGGTG
pilE1-R2	GCCAAAGCAGTCGGATAAGA
pilE2-F1	TGCAGGTGCTGAAACAAATC
pilE2-R1	GGTGATATTCTCATTTTAGCCATATGTATTTTCTCCTAATGTTTTTATG
pilE2-F2	TTTTACTGGATGAATTGTTTTAGACCGAAGCTGGTGCTAATTC
pilE2-R2	GTGCCGATCTCAAATGTCCT
pilA-F1	ATTATCGCCATCATCGCAGC
pilA-R1	GGTGATATTCTCATTTTAGCCATTGATAATATCTCCTAGTTTTCTCTTTTG
pilA-F2	TTTTACTGGATGAATTGTTTTAGCACAAAAGGAATTCGCTCGGA
pilA-R2	TCCCTGCAAATCCCAATCCA
pilB-F1	TACAACTGGACCGAAGCTGG
pilB-R1	GGTGATATTCTCATTTTAGCCATCATTCCTACCTATTTATTTTTACTTCTG
pilB-F2	TTTTACTGGATGAATTGTTTTAGAGGATTTGTGGTTTGTATCAGGG
pilB-R2	TTTGGCCTATCGTTCCCACT
pilC-F1	GTGGGCTTCTCTACTTCAGC

pilC-R1	GGTGATATTCTCATTTTAGCCATCTCTGCTCCCTTCTACGGC
pilC-F2	TTTTACTGGATGAATTGTTTTAGCTTCCGCGATAAGTATAAGGCA
pilC-R2	CACTAGCTGCCACATCAACC
pilM-F1	CACTTGATGGCCGTACGATG
pilM-R1	GGTGATATTCTCATTTTAGCCATAATCTCCTAATCTCCCTTATTTTTCC
pilM-F2	TTTTACTGGATGAATTGTTTTAGGGAGTTCATTCCGGCTACAG
pilM-R2	GATACCCTCCACCAGCAAGT
pilN-F1	ATCTGGGCTTGGCTGAGAAT
pilN-R1	GGTGATATTCTCATTTTAGCCATTAGTTCGCTCTCCTAATCAACATG
pilN-F2	TTTTACTGGATGAATTGTTTTAGAACATTAAAATTTTGACGAAGGATG
pilN-R2	CTGCTGCTTGTTGACGATGA
pilH-F1	TTCTGCCGAGTTTAATGCGG
pilH-R1	GGTGATATTCTCATTTTAGCCATCTCATTTTTGCTCTTCATTGAAATC
pilH -F2	TTTTACTGGATGAATTGTTTTAGCCTGAAACGACTATGAAGCTGG
pilH-R2	GGCACAGAGTCCAACAAAGG
pilI-F1	AATGGAATTTGCAGCACCGT
pilI-R1	GGTGATATTCTCATTTTAGCCATTTTCTAAATGATATTAAACTTTTTCAATGC
pilI-F2	TTTTACTGGATGAATTGTTTTAGTCAAGAAGCGTTCAACATGC
pilI-R2	ACGGCCCATCTCTACATCTG
pilJ-F1	ATGGATTCCGCCTTCAGTCA
pilJ-R1	GGTGATATTCTCATTTTAGCCATAATTTCCCCTAAAACCCTTCTAATTG
pilJ-F2	TTTTACTGGATGAATTGTTTTAGGCCGTATCGACCGTAAATTC
pilJ-R2	ATTGGTCGTTGGAAGCTGAC
pilK-F1	CAGATGTAGAGATGGGCCGT
pilK-R1	GGTGATATTCTCATTTTAGCCATGAATCTCATCCCAACCTTATACTACG
pilK-F2	TTTTACTGGATGAATTGTTTTAGATTAAGTTTGGAGGAGGGGC
pilK-R2	TGTAATAAGCCTTGGCCCCA
pilD-F1	CCGTTTTTCGATACCAAGGA
pilD-R1	GGTGATATTCTCATTTTAGCCATAATTTTTCCCTTTTTATACTCTTTGAA
pilD-F2	TTTTACTGGATGAATTGTTTTAGGAGTATCATGGCGGTCATCC
pilD-R2	TTTAGAGCCCCAAAGAGCAA
2229-F1	TGCCAAAGGTCGGTCTATGT
2229-R1	GGTGATATTCTCATTTTAGCCATTTTTTCTATCCATTTCTATTGTCGCTT

2229-F2	TTTTACTGGATGAATTGTTTTAGTGGTCAAGCAAGATTATGATTCG
2229-R2	CCAACAGCCCTCCAGAAGTT
2228-F1	GCCAAGAAAACAGGAGCAGT
2228-R1	GGTGATATTCTCATTTTAGCCATAAAAGACGATGTCGCAGAAGC
2228-F2	TTTTACTGGATGAATTGTTTTAGATCCTGATGTCTTATGATCGGG
2228-R2	TCACAATGGCCTCTGGACTT
2227-F1	AACTTCTGGAGGGCTGTTGG
2227-R1	GGTGATATTCTCATTTTAGCCATCGTTCATTATCCTAAATCCTAAAACCC
2227-F2	TTTTACTGGATGAATTGTTTTAGGCCGATACTCTGATGGAAAATG
2227-R2	ACCCAATGCAATCCCCAAAC
2226-F1	ATTTTGGCTATGCGGTTTGG
2226-R1	GGTGATATTCTCATTTTAGCCATATCCTTCCTCTTGTTTCTATCAA
2226-F2	TTTTACTGGATGAATTGTTTTAGGTCAAAACCTTCAACGAAGC
2226-R2	TCAATAACGAGGCCAACTCC
2225-F1	CCCTCTGGTGGACTCATGTT
2225-R1	GGTGATATTCTCATTTTAGCCATGATAGACTCCTTTCCTTATGTTAGAAAT
2225-F2	TTTTACTGGATGAATTGTTTTAGTGTCTATCTTATCTTGGAGCGGA
2225-R2	GCTCTCACCGTGTATCCCTT
2224-F1	AACGGGTGGCATCATTACAA
2224-R1	GGTGATATTCTCATTTTAGCCATGGTTATCCTCTCCGCTCCAA
2224-F2	TTTTACTGGATGAATTGTTTTAGCTTTGAACTCAGACAGAAAGGGG
2224-R2	TACACATGATCCCCAGCCAG
comGB-F1	ACTGTGAGGAAGCCAAGGTT
comGB-R1	GGTGATATTCTCATTTTAGCCATTGATATGTCCTTCTGCAAAAAG
comGB-F2	TTTTACTGGATGAATTGTTTTAGTGTTTTACTTTATGCGGCAATGC
comGB-R2	GTCCTGTCCTCCGTCTGAAA
rpsl-F	GGCAGGTGTAGCTGTCTTTG
rpsl-R	CTCTTGCTCCATCCAGTCCA

Regions of complementarity for splicing PCR are underlined.

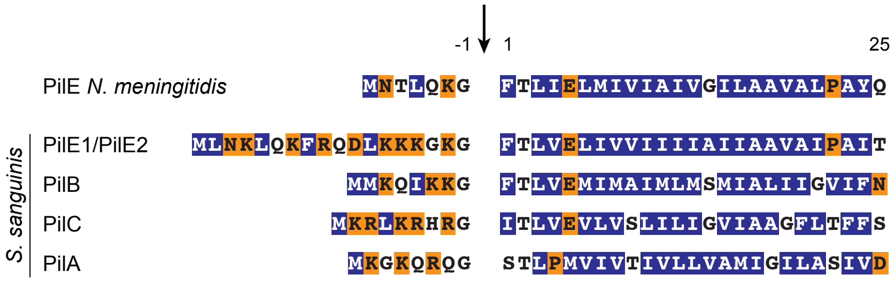

Fig. S1. Five proteins in the *pil* locus of *S. sanguinis* 2908 harbour a putative class III signal peptide. The N-terminal sequence of a prototypical type IV a pilin, *N. meningitidis* PilE, is shown for comparison. The five *S. sanguinis* proteins (the N-termini of PilE1 and PilE2 are identical and therefore only one sequence is displayed) contain 8-18 aa long leader peptides, which contain a majority of hydrophilic (orange) and neutral (no shading) residues. This leader peptide ends with a conserved Gly after which processing by the prepilin peptidase (indicated by a vertical arrow) occurs. The mature proteins start with a tract of 21 predominantly hydrophobic residues (shaded in blue), which is expected to form an extended α-helix that is the main assembly interface within filaments. Unlike the other four proteins, PilA has a degenerate class III signal peptide since the first residue of the mature protein is a Ser rather than a Phe, and the usually conserved negatively charged Glu_5 residue is replaced by a Pro_4.

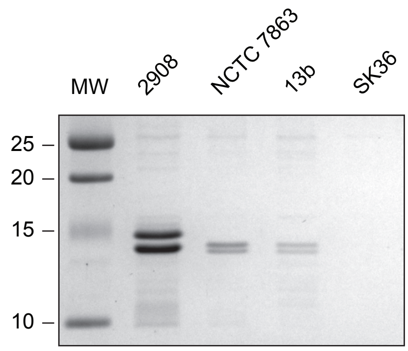

Fig. S2. Tfp of several *S. sanguinis* isolates are composed of multiple pilins. Tfp were prepared from mid-logarithmic phase cultures of NCTC 7863, 13b and SK36. The 2908 strain was included as a positive control. Cultures were adjusted to the same OD_{600}, separated by SDS-PAGE and stained with Coomassie blue. Identical volumes were loaded in each lane. Molecular weights are indicated in kDa.

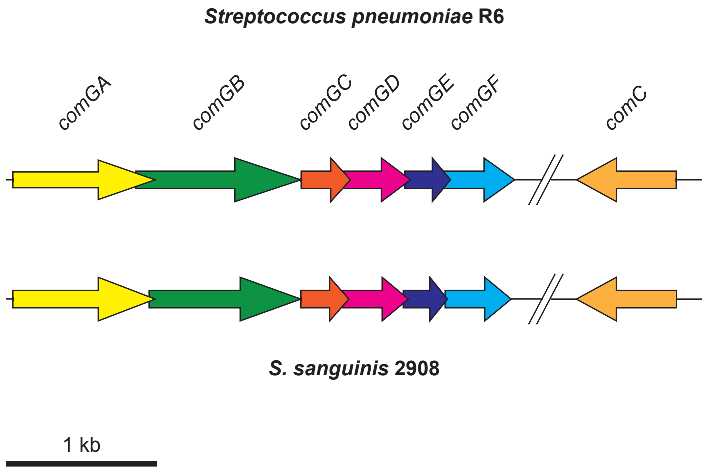

Fig. S3. Comparison of the genes encoding the competence (pseudo)pilus in *Streptococcus pneumoniae* R6 and *S. sanguinis* 2908. All the genes are drawn to scale, with the scale bar representing 1 kb. Genes encoding orthologous proteins are coloured the same.

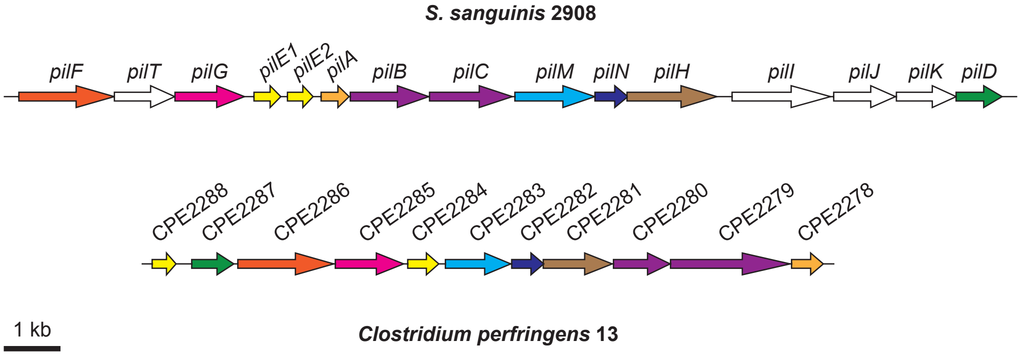

Fig. S4. Comparison of the *pil* loci in *S. sanguinis* 2908 and *Clostridium perfringens* 13. All the genes are drawn to scale, with the scale bar representing 1 kb. Genes encoding proteins predicted to play similar functions are coloured the same.

Supplementary movies legends

Movie S1. Cellular motility exhibited by *S. sanguinis* 2908. A small chain of cells attached to a coverslip was imaged for 30 sec. The scale bar represents 5 µm.

Movie S2. Δ*pilT* cells exhibit only residual Brownian motion. A small chain of cells attached to a coverslip was imaged for 30 sec. The scale bar represents 5 µm.

Movie S3. Deflection of the top of PoMPs resulting from pulling events by *S. sanguinis* 2908. A small microcolony, in the middle of the field, on top of a regular array of PoMPs was imaged for 100 sec.

Movie S4. PoMPs in contact with a Δ*pilT* mutant show no visible deflection, indicative of an absence of pulling forces. A big microcolony, in the middle of the field, on top of a regular array of PoMPs was imaged for 100 sec.

Movie S5. Cellular motility exhibited by a Δ*pilK* mutant. A small chain of cells attached to a coverslip was imaged for 30 sec. The scale bar represents 5 µm.
